# Death Anxiety and Depression in Amyotrophic Lateral Sclerosis Patients and Their Primary Caregivers

**DOI:** 10.3389/fneur.2018.01035

**Published:** 2018-12-03

**Authors:** Marvin R. Grabler, Ute Weyen, Georg Juckel, Martin Tegenthoff, Paraskevi Mavrogiorgou-Juckel

**Affiliations:** ^1^Department of Psychiatry, Psychotherapy and Preventive Medicine, LWL-University Hospital of Ruhr-University Bochum, Bochum, Germany; ^2^Department of Neurology, Bergmannsheil University Hospital of Ruhr-University Bochum, Bochum, Germany

**Keywords:** amyotrophic lateral sclerosis, motor neuron disease, ALS-FRS, death anxiety, BOFRETTA, caregivers

## Abstract

**Background:** Given the lethal severity of amyotrophic lateral sclerosis (ALS), the aim of this study was to illuminate the coherence of depression and death anxiety in both ALS patients and caregivers and in how far patients and caregivers are influenced by the mindset of their respective counterpart.

**Methods:** 30 couples of patients (mean age 60.57; 13 women, 17 men) and primary caregivers (mean age 57.33; 16 women, 14 men) were included into the study. Death anxiety was assessed using the newly developed BOFRETTA scale, depression via Beck Depression Inventory, anxiety by means of State Trait Anxiety Inventory and caregivers' exertion using the Caregiver Strain Index. Patients' impairment was assessed with the ALS functional rating scale.

**Results:** We found that while death anxiety was related to depression in both patients and caregivers, death anxiety was related to anxiety only in patients. Caregiver strain correlated with both caregiver‘s depression and anxiety. Moreover, patients' and caregivers’ depression, anxiety and death anxiety correlated to the ones of their counterpart.

**Conclusion:** These results suggest that despite little depressive symptoms in ALS patients the fatal prognosis of the disease takes into account, depression and death anxiety influence each other and might be addressed together in pharmacological and especially psychotherapeutic interventions to the benefit of the patient. Medical professionals should not forget to offer sufficient support to caregivers tending patients affected by depression and death anxiety as they are likely to mirror their patient's feelings.

## Introduction

Amyotrophic lateral sclerosis (ALS) is the most common motor neuron disease with a prevalence of 6 cases per 100.000 persons, whereas the incidence is approximately 1–2 cases/100.000 persons (1). With rapidly progressive degeneration of both first and second motor neuron, ALS has a mean mortality rate of 50% in just 3 years (2). Despite this severe prognosis, clinical depression is less frequent than one might expect (3) with different studies suggesting rates of 9–12% (4–6).

Thus, depression is less common in ALS patients than in patients suffering from other diseases of comparable impact on patients' lives and agility like multiple sclerosis (7) or cancer (8), although death is mostly less imminent in these diseases. Anxiety, e.g., measured with the State-Trait Anxiety Inventory (STAI), amounts to likewise low levels albeit it is much less frequently studied (9). Furthermore, no consistent association between depression as well as other psychopathological impairments and wish to die in ALS patients were found (5, 6, 10). In how far defense mechanisms like denial come into effect here, is unknown. Some studies admit the conclusion that at least in the terminal phase such mechanisms play a role to make the situation endurable since the degree of death anxiety is related to the denial of one's own finiteness (11). The conversion of death anxiety into situational anxiety limits the perception of one's own finiteness and has therefore been stated as a coping strategy (12). Failure of such coping strategies may serve as a catalyst for the formation of mental disorders like depression as pathologic dimensions of death anxiety have been discussed to relate to other mental disorders, e.g., anxiety disorders (13). The few studies touching the subject of anxiety in ALS patients suggest that anxiety in general and thus presumably also death anxiety mostly occur in early stages of the disease and the diagnostic phase and to lesser extent in the course of the disease which is marked by progressive bodily impairment (14–17). It is yet left for speculation whether the little signs of depression and anxiety in the majority of ALS patients, that might appear like acceptance of the disease to outsiders, are a sign of a coping strategy of whatever nature, are caused by the cerebral alterations in cognition and emotion, which take place even in early stages of ALS (18), or have yet another explanation.

Taking into account the rapidly progressing impairment that makes patients dependent on assistance and thus exacts a close daily contact between patient and caregiver, it appears inevitable to evaluate on the one hand in how far and under which circumstances this relation acts protective or may lead to the exacerbation of the surveyed values of depression and anxiety in the patient and on the other hand the effect on the caregiver of the patient's state of mind and vice versa. Studies involving caregivers of ALS patients have shown that a sophisticated evaluation of quality of life, coping strategies, and anxiety are pivotal to develop suitable therapy and relief efforts (15). More than 30% of caregivers of ALS patients have been reported to indicate a marked worsening of their situation with a reduced quality of life (19). A somatic affection of caregivers has likewise been reported several times (18, 20), but this appears to be related more to levels of depression of the relative suffering from ALS (21), which underlines once more the interrelation between caregiver and patient.

Considering the hardly comprehensible reaction of ALS patients to their severe disease in the sense of few depressive symptoms and anxiety, the aim of this study was therefore to further illuminate the ties of depression, anxiety and death anxiety with respect to the setting of a close contact of ALS patient and caregiver.

We hope that our findings will further facilitate the establishment of adequate support measures and therapies for the coping of severe diseases like ALS for patients themselves and their caregivers in the interest of mental health.

## Materials and methods

We interviewed a succession of 30 couples of patients and their primary caregivers seen in our outpatient clinic specializing in ALS. The patients, commonly accompanied by their caregiver, are seen at regular intervals of about 3–4 months on average for evaluation of disease development and assistive device need. Inclusion criteria consisted of ALS diagnosis in accordance with revised El Escorial criteria (22) and fluency in German.

Table 1 shows the socio-demographic and clinical data of the 30 patients and their caregivers being included in the study. Most patients were receiving a variety of constant medications including riluzole (80%) agent.

**Table 1:** Socio-demographic and clinical characteristics of patients (PAT) and caregivers (CG).

	**PAT (*n* = 30)**	**CG (*n* = 30)**	***t*-Test**
**GENDER**			
Female, *n*	13 (43.3%)	16 (53.3%)	
Male, *n*	17 (56.7%)	14 (46.7%)	
**AGE**			
Mean (*SD*)	60.57 (8.52)	57.33 (9.47)	
Range	44–78 years	38–75 years	
**FAMILY STATUS**			
Married/with partner, *n*	26 (86.7%)	29 (96.7%)	
Single, *n*	1 (3.3%)	0	
Divorced/widowed, *n*	3 (10%)	1 (3.3%)	
**EDUCATIONAL BACKGROUND**			
13 years of school, *n*	7 (23.3%)	10 (33.3%)	
10 years of school, *n*	13 (43.3%)	12 (40%)	
9 years of school, *n*	10 (33.3%)	6 (20%)	
Did not graduate, *n*	0	2 (6.7%)	
**EMPLOYMENT STATUS**			
Employed, *n*	8 (26.7%)	18 (60%)	
Retired, *n*	11 (43.7%)	9 (30%)	
Retired due to ALS, *n*	8 (26.7%)	–	
Unemployed/homemaker	3 (10%)	1 (3.3%)	
Other/did not answer	0	2 (6.6%)	
**CLINICAL SCORES**			
ALS-FRS mean (*SD*)	32.57 (9.51)	–	
Disease duration (*SD*), Years	2.48 (2.0)	–	
Hours/day spent with caring (*SD*)	–	3.37 (2.85)	
Affection of own health	–	10 (33.3%)	
BDI mean (*SD*)	11.93 (8.40)	8.07 (8.08)	ns
STAIs mean (*SD*)	40.67 (12.5)	45.67 (11.62)	ns
CSI, mean (*SD*)	–	4.67 (3.48)	
BOFRETTA anxiety (*SD*)	25.97 (9.85)	23.5 (8.33)	ns
BOFRETTA attitude (*SD*)	17.43 (3.78)	16.33 (4.05)	ns

*ALS-FRS, ALS Functional Rating Scale; BDI, Beck Depression Inventory; STAIs, State-Trait Anxiety Inventory, state subscale; CSI, Caregiver Strain Index; BOFRETTA, Bochumer Fragebogen zur Einstellung zum Tod und zur Angst vor dem Tod (Bochum Questionnaire regarding Attitude toward and Anxiety for Death), subscales anxiety and attitude; ns, non-significant*.

All participants gave written informed consent after the study purpose was explained in detail. The ethics committee of the Medical Faculty of the Ruhr-University Bochum, Germany, approved the study. It was carried out in accordance with the Declaration of Helsinki of 1975.

### Clinical assessment

Severity of depressive symptoms was assessed using the Beck Depression Inventory [BDI; (23)], which generates a score from 0 to 63 with 0–12 indicating no depression, 13–19 mild, 20–28 medium, and ≥29 severe depression.

State anxiety was examined using the State-Trait Anxiety Inventory. The score ranges from 20 to 80 with 20–39 indicating low, 40–59 medium, and ≥60 high anxiety (24, 25).

The Caregiver Strain Index (CSI) by Robinson (26) on the other hand is a questionnaire with 13 items to be answered with either Yes or No, which depicts the caregivers strain (e.g., due to loss of sleep, changes in private life or bothering behavior). The CSI was only given to the caregivers.

For the evaluation of death anxiety in particular we utilized the BOFRETTA-Scale (27) for the first time, containing 25 statements about attitude toward (10 items) and anxiety for (15 items) death. The scale is based on or taking cue for its modification from Templer's death anxiety scale (28), Lester's death anxiety scale (29) and the question inventory for multi-dimensional evaluation of experience of dying and death [FIMEST; (30)]. To answer the semiquantitative BOFRETTA-scale one can indicate one's accordance with the statements by choosing between “does not apply at all” (1 point), “applies slightly” (2 points), “applies predominantly” (3 points), or “applies mostly” (4 points), amounting to scores from 10 to 40 for the subscale “attitude toward death” (items number 2, 3, 10, 16, 17, 19, 21–23, 25, and 15–60 for the subscale “death anxiety” (items number 1, 4–9, 11–15, 18, 20, 24). As qualitative analysis, the participant may express personal thoughts or concerns, respectively toward death in two free text columns.

The patient's physical impairment due to the disease was assessed by the ALS functional rating scale [ALSFRS-R; (31)] which reaches from 48 (no impairment at all) to 0 (locked-in syndrome), which is routinely taken in the outpatient clinic.

### Statistics

Further statistical analyses of the neuropsychological data were performed using IBM SPSS Statistics for Windows, Version 25.0 (IBM Corp., Armonk, NY, USA). Statistical analyses were performed with appropriate parametric or non-parametric tests (*t*-test and Pearson or Spearman correlation coefficients). Statistical significance was defined as *p* < 0.05. A value of *p* < 0.10 was regarded as statistical tendency.

## Results

### Patients

The socio-demographic characteristics as well as psychometric scores of the 30 patients interviewed (17 men and 13 women) are summarized in Table 1. The patients had a mean age of 60.57 years (*SD* 8.52) and suffered from ALS for 2.48 years (*SD* 2.0) on average. The mean ALSFRS-R was 32.57 (*SD* 9.51).

The mean BDI score amounted to 11.93 (*SD* 8.40), signifying a score which is just still within the non-depressive range. 18 (60%) of the patients interviewed were in the non-depressive range according to BDI, while 9 (30%) had mild, 2 (6.7%) medium, and just 1 (3.3%) patient severe depression.

As for the STAI, the average patient score was 40.67 (*SD* 12.5), lying just inside of the medium anxious range. Nevertheless, the biggest group of 15 patients (50%) showed low signs of anxiety, while 12 (40%) were in the medium range and 3 (10%) in the high range.

When assessed by means of the BOFRETTA death anxiety subscale the average patient score was 25.97 (*SD* 9.85). Taking into account the range from minimum 15 to maximum 60, this is a mean score within the lower third. Anyhow, the maximum score of any patient interviewed was 54. Similar patterns apply to the BOFRETTA attitudes toward death subscale with a possible range from 10 to 40, where we found a mean patient score of 17.43 (*SD* 3.78), thus not particularly negative.

### Caregivers

The mean age of the 30 interviewed caregivers (16 women and 14 men) was 57.33 years (*SD* 9.47). For a summary of socio-demographic and psychometric parameters see Table 1 again.

The average BDI score of caregivers was 8.07 (*SD* 8.08), which is more clearly within the non-depressive range compared to patients. Accordingly, 24 (80%) of caregivers achieved non-depressive scores, while 4 (13.3%) had mild, and each 1 caregiver (3.3%) had medium or severe depression.

45.67 (*SD* 11.62) was the mean STAI score, making it the only parameter caregivers scored higher than patients (though the difference is statistically non-significant). This is within the medium anxious range. 12 (40%) of caregivers showed low, 16 (53.3%) medium, and 2 (6.6%) high signs of anxiety.

BOFRETTA anxiety subscale amounted to 23.5 (*SD* 8.33) on average and BOFRETTA attitude subscale to 16.33 (*SD* 4.05)—both scores likewise within the lower third.

Finally, the CSI resulted in a mean 4.67 (*SD* 3.48), which can be classified on the verge of low to medium third of caregiver strain, given that the scale ranges from 1 to 13. The highest CSI score found in our study was 11. The caregivers spent 3.37 h a day (*SD* 2.85) on average caring for their relative. 10 (33.3%) of caregivers reported an affection of their own health due to caring. Most stated psychic symptoms (stress, difficulties to concentrate or to sleep, feeling “burnt out,” tension) and just two somatic symptoms were stated (dorsal pain, irritable bowel syndrome).

### Comparison of couple scores

Sociodemographic parameters as well as psychometric scores did not differ significantly between patients and caregivers.

We found various correlations between scores of patients and caregivers (see Table 2): Patients' and Caregivers' depression related to the ones of their particular counterpart highly significantly, just like patients' and caregivers' anxiety. While patients' depression did not relate to caregivers' anxiety, patients' anxiety related to caregivers' depression, indicating a specific pattern of reaction on either side to the perceived emotions of one's relative.

**Table 2:** Comparison of couple scores.

**Patient score**	**Caregiver score**	**Correlation coefficient**
BDI	BDI STAIs CSI	0.590^**^ ns 0.373^*^
STAIs	STAIs BDI	0.516^**^ 0.708^**^
BOFRETTA anxiety	BOFRETTA anxiety BDI	0.375^*^ 0.466^*^
BOFRETTA attitude	BOFRETTA attitude	ns
Years with ALS	CSI	ns
ALS-FRS	CSI BDI STAIs BOFRETTA anxiety BOFRETTA attitude	−0.629^**^ ns ns ns ns

*ALS-FRS, ALS Functional Rating Scale; BDI, Beck Depression Inventory; STAIs, State-Trait Anxiety Inventory, state subscale; CSI, Caregiver Strain Index; BOFRETTA, Bochumer Fragebogen zur Einstellung zum Tod und zur Angst vor dem Tod (Bochum Questionnaire regarding Attitude toward and Anxiety for Death), subscales anxiety and attitude; ns, non-significant*;

* *Statistically significant*;

** *Statistically highly significant*.

While death anxiety of patients and caregivers correlated with each other, this was not the case for attitudes toward death. Besides, patients' death anxiety and caregivers' depression related to each other.

The years of patients has already lived with ALS has no relation to the caregiver's strain. The ALSFRS-R relates negatively to the CSI, thus meaning higher bodily impairment imposes more caregiver strain. Nevertheless, the ALSFRS-R does not relate to caregivers' depression, anxiety, death anxiety, or attitudes toward death.

### Psychometric correlations

Correlations of psychometric and clinical parameters are summarized in Table 3.

**Table 3:** Psychometric correlations.

**Scores**		**Patient (*n* = 30)**	**Caregiver (*n* = 30)**
		**Correlation coefficient**	**Correlation coefficient**
ALS-FRS	BDI	−0.374^**^	–
STAI	BDI	0.652^**^	0.676^**^
STAI	BOFRETTA anxiety	0.510^**^	ns
STAI	BOFRETTA attitude	0.373^*^	ns
BOFRETTA anxiety	BDI	0.394^*^	0.432^*^
BOFRETTA anxiety	BOFRETTA attitude	0.577^**^	0.744^**^
BOFRETTA attitude	BDI	0.414^*^	ns
CSI	BDI	–	0.409^*^
CSI	STAI	–	0.412^*^
CSI	Hours/day caring	–	0.729^**^
Hours/day caring	BDI	–	0.444^*^
Own health affection	STAI	–	0.413^*^

*ALS-FRS, ALS Functional Rating Scale; BDI, Beck Depression Inventory; STAIs, State-Trait Anxiety Inventory, state subscale; CSI, Caregiver Strain Index; BOFRETTA, Bochumer Fragebogen zur Einstellung zum Tod und zur Angst vor dem Tod (Bochum Questionnaire regarding Attitude toward and Anxiety for Death), subscales anxiety and attitude; ns, non-significant*;

* *Statistically significant*;

** *Statistically highly significant*.

Anxiety and depression scores correlated highly significantly to each other for both patients and caregivers. Death anxiety correlated with negative attitudes toward death and depression.

In patients, ALSFRS-R correlated negatively to BDI scores. As low ALSFRS-R scores reproduce high bodily impairment due to the disease, this indicates that patients in a worse physical condition are more prone to depression. Anxiety correlated to death anxiety and negative attitudes toward death; negative attitudes toward death correlated to depression.

Taking a closer look at the caregivers, we found that caregiver strain as screened by CSI correlated with depression, anxiety and the amount of hours spent caring per day. The amount of hours spent caring also correlates with depression, while the affection of the caregiver's own health correlates with anxiety.

## Discussion

Integrating our findings into the relevant literature, we found rates for depression of patients on similar low levels (4–6), even if only taking into account studies likewise using the BDI to assess depression (9) as different diagnostic tools sometimes lead to varying results. Patients also seem to be not particularly anxious mirroring findings of Vignola et al. (15) for patients a while after diagnosis.

Through ALSFRS-R, we were able to correlate more severe neurological impairment to higher rates of depression, which has also been shown before (5). We could not find a direct relation of neurological impairment to anxiety nor death anxiety in particular. As rates of death anxiety are not high in ALS patients in general according to our findings, one might ascribe it to the fact that views on death are very individual: Just because of increasing health issues, one does not have to become anxious about death as patients might have positive attitudes toward death, may it be due to religious beliefs or even judging death as a kind of salvation—patients may become depressed due to the impact the disease has on their daily lives and activities. Many patients are able to find meaning in life despite their disease (14), which makes them able to cope with anxiety and death anxiety better.

We found noteworthy relations of depression, death anxiety and anxiety. While depression and anxiety correlates for both patients and caregivers, death anxiety relates to anxiety and negative attitudes toward death only in patients. This is easily explained taking into account that the own death is more imminent for patients, thus likely more often on their mind, making it a probable central aspect of anxiety. With respect to the fact that depression correlates with death anxiety in both patients and caregivers this circumstance, which may appear self-explanatory at first, gets a therapeutic impact: If death anxiety is present, it forms a sort of continuum with depression and anxiety in patients. Fighting depression, professionals might benefit from broaching the issue of death and death anxiety with the patients affected and giving room to talk about the feelings associated. In the largely agnostic societies in Western Europe, persons so suddenly confronted with the finiteness of their own lives, may ask questions they yet never thought about or pushed away. If the patient is open to it, health care professionals might even think about establishing contact to spiritual counsellors—company which is almost standard in hospices but rather not in ambulant palliative care.

The low rates of depression and anxiety we found in patients also apply to caregivers though anxiety seems to be slightly more widespread in caregivers. Albeit the difference is non-significant, as similar findings occurred in another study (15) the question how to relieve the caregivers from their anxiety becomes ever more important. Since there is no relation to death anxiety according to our data, the anxiety might rather be focused on the current situation: e.g., on the development of the loved one's disease and whether the own person will be able to fulfill the caregiving role further onward. These concerns should thus be tackled with sufficient support in the form of auxiliary means for the patient and perhaps even the hiring of professional nursing services.

A low ALSFRS-R as a measure of disease severity does not relate to caregiver's depression or anxiety, signifying that there are caregivers and patients who cope well despite vast progression of the disease. Nevertheless, caregiver strain derives from the severity of ALS as such, own depression and anxiety and also patient's depression. Notwithstanding, higher caregiver strain does not result in more negative attitudes toward death as we hypothesized.

The striking, highly significant relation of depression, anxiety and death anxiety of patients, and caregivers in our study suggests that the patient-caregiver-relation is of huge importance for coping with ALS. As evident from our data and surely noticeable while conducting the interviews, there were couples of patient and caregivers who found ways to deal with the disease and live merely content despite the changes in their daily lives. Other couples were struggling more. If the patient was depressed, the caregiver was quite likely to mirror these feelings and vice versa. This further underlines the importance of keeping an eye on the patient's surroundings and potentially offering medical or psychotherapeutic assistance to the caregiver as well. Likewise, in psychotherapeutic interventions targeting at depression, the patient-caregiver relation possibly ought to be included into the therapeutic sessions and play a pivotal role.

Limitations of our study include a rather small sample size, though this seems to be universal for ALS research. Methodologically, one may criticize the use of questionnaires not entirely suitable for ALS patients with e.g., BDI asking for weight loss which most likely is not a sign of depression but muscle loss in ALS patients and thus overestimating depressive rates. BDI is a well-established device and ALS-specific questionnaires would have made the comparison to caregivers impossible. Apart from that, some patients were treated with antidepressants.

In conclusion, although depression, anxiety, and death anxiety are not particularly common in ALS patients, we found that they widely correlate with each other and should be addressed altogether. Furthermore, the relation of patient and caregiver and their respective mind-sets play a significant role in coping with the disease and therefore should be considered in medical and psychotherapeutic interventions. After all, even a devastating diagnosis like ALS does not inevitably lead to depression and anxiety—we met many resilient couples who positively deal with their situation and find meaning in life.

## Ethics statement

The ethical commission of Faculty of Medicine of RUB has approved this study under the correspondence number 15/5384 (23.3.17).

## Author contributions

PM-J, MT, and GJ designed this study. MG and UW have performed recruitment and assessment of patients and caregivers. MG and PM-J have analyzed the data and wrote the first draft. All authors have approved the final version. This manuscript is part of the doctoral thesis of MG.

### Conflict of interest statement

The authors declare that the research was conducted in the absence of any commercial or financial relationships that could be construed as a potential conflict of interest.
